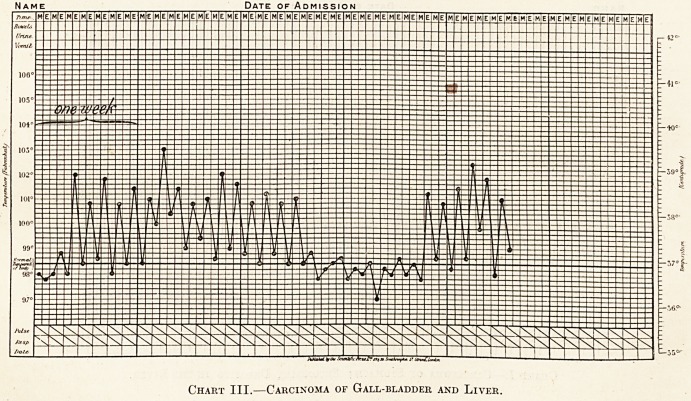# Pyrexia in Malignant Disease of the Liver

**Published:** 1907-11-16

**Authors:** 


### Clinical Points.

### PYREXIA IN MALIGNANT DISEASE OF THE LIVER.

We have already discussed the question of pyrexia in association with cirrhosis of the liver, and we drew attention to the fact, first noted by Carrington, that such pyrexia is the rule rather than the exception. Cirrhosis, however, is by no means the only non-suppurative affection of the liver that commonly causes some degree of fever. Malignant disease does so too in the majority of cases, and the temperature charts of patients with hepatic cirrhosis are closely simulated by those of patients suffering from carcinomatous deposits in the liver.

Why this should be so we do not clearly know. The liver, of course, is a very vascular organ, containing something like one-quarter of the blood that is present in the entire body; it therefore affords a very large surface for the absorption into the blood stream of inflammatory or other abnormal products that are being formed in it. Both in cases of cirrhosis and in those in which there are multiple deposits of growth in the organ, the degree of inflammation at any one spot in the liver is usually very slight; but when small amounts of abnormal chemical products are being absorbed from a large number of these foci, the net result is that the blood, as it leaves by the hepatic veins, is charged with a large quantity of these products. What the products are we do not know, and therefore we call them toxins; it is quite conceivable that these unknown chemical substances absorbed from cirrhotic and malignant livers are the same as those derived from ordinary inflammations elsewhere; but whereas in other places, if pyrexia results, the degree of inflammation is usually little short of pus-formation, in the case of the liver pyrexia can ensue from the concentration together in the blood of the products of multiple foci of comparatively slight inflammation.

Whether the explanation suggested is the true one or not, the fact remains that even considerable pyrexia may occur in cases of malignant disease of the liver without there being any suppuration at all. This is of considerable clinical importance, for if it were not recognised it might be thought that the pyrexia indicated the necessity for surgical assistance—an empyema of the gall-bladder might be supposed to exist, or something of that sort; or it might be feared that lobar pneumonia or some similar complication had set in. It is, of course, possible that pus-formation or some intercurrent affection may occur in cases of malignant disease of the liver, but the present point is that the existence of pyrexia is not by itself a proof of this. When a number of cases are investigated, it is found that about one-third are apyrexial, whilst about two-thirds have pyrexia. As in cirrhosis, so in malignant disease of the liver, the temperature is usually normal in the morning, but rises in the evening, and in the majority of cases the rise is moderate, up to 99° F. or 100° F., as in the following typical chart:—

Chart I.—Carcinoma of Stomach; Secondary Deposits in the Liver.

In a smaller number of cases the rise may be greater than this, and although the tendency always is for the temperature to approach normal in the morning, it sometimes fails to reach normal every day, as in Chart II.

This chart shows one point to which Dr. J. W. Russell has drawn attention—namely, that there are periods of remission from time to time; and such periods of remission may occur even when the pyrexia before and after such a period is considerable, as in Chart III. :—

Chart II.—Carcinoma of the Colon; Secondary Deposits in the Liver.

Chart III.—Carcinoma of Gall-bladder and Liver.

The above charts must only be regarded as instances for the degree of pyrexia varies considerably. The important point to recognise is that the pyrexia can, and usually does, occur when the liver is the seat of malignant disease. The primary growth may be in the gall-bladder, or it may be in some distant part, such as the rectum. It is the metastatic deposits in the liver which seem to cause the pyrexia, and they do so, as we have explained, without there being any macroscopic degree of purulent infection. It is worthy of note, perhaps, that the pyrexia in these cases is unassociated with rigors and that the pyrexia is as liable to be present with sarcomatous as with carcinomatous deposits in the liver.

## Figures and Tables

**Chart I. f1:**
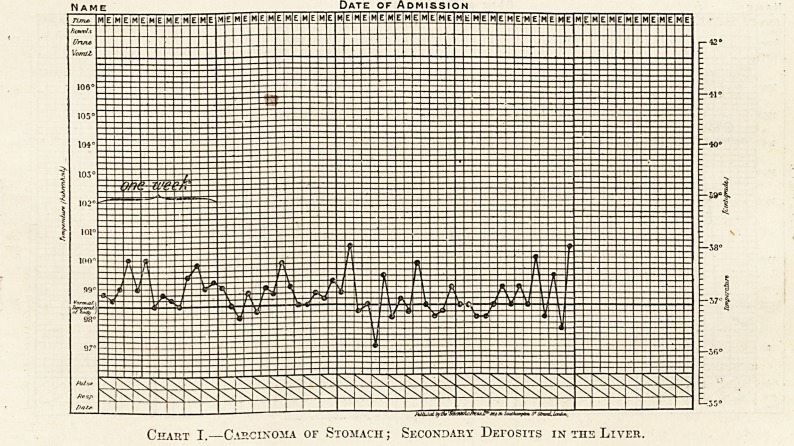


**Chart II. f2:**
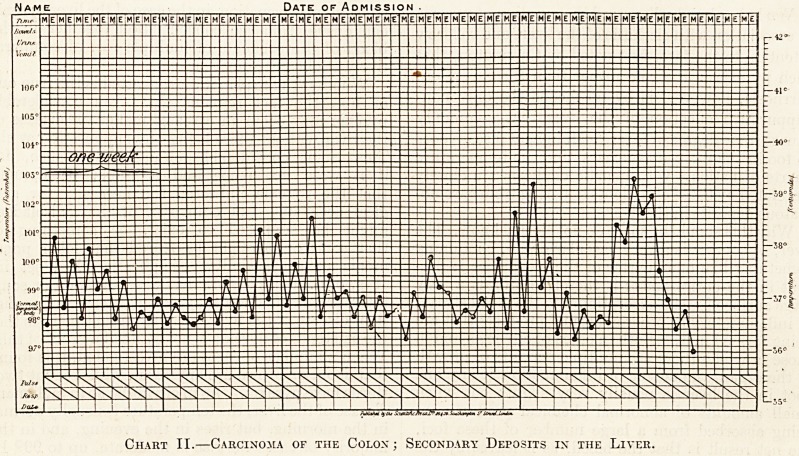


**Chart III. f3:**